# Production of xylooligosaccharides and monosaccharides from poplar by a two-step acetic acid and peroxide/acetic acid pretreatment

**DOI:** 10.1186/s13068-019-1423-x

**Published:** 2019-04-15

**Authors:** Peiyao Wen, Tian Zhang, Jinye Wang, Zhina Lian, Junhua Zhang

**Affiliations:** 1 College of Forestry, Northwest A&F University, 3 Taicheng Road, Yangling, 712100 Shaanxi China; 2 Co-Innovation Center for Efficient Processing and Utilization of Forest Products, College of Chemical Engineering, Nanjing Forestry University, Nanjing, 210037 Jiangsu China

**Keywords:** Poplar, AC pretreatment, HPAC pretreatment, Lignin removal, Xylooligosaccharides, Enzymatic hydrolysis

## Abstract

**Background:**

*Populus* (poplar) tree species including hybrid varieties are considered as promising biomass feedstock for biofuels and biochemicals production due to their fast growing, short vegetative cycle, and widely distribution. In this work, poplar was pretreated with acetic acid (AC) to produce xylooligosaccharides (XOS), and hydrogen peroxide–acetic acid (HPAC) was used to remove residual lignin in AC-pretreated poplar for enzymatic hydrolysis. The aim of this work is to produce XOS and monosaccharides from poplar by a two-step pretreatment method.

**Results:**

The optimal conditions for the AC pretreatment were 170 °C, 5% AC, and 30 min, giving a XOS yield of 55.8%. The optimal HPAC pretreatment conditions were 60 °C, 2 h, and 80% HPAC, resulting in 92.7% delignification and 87.8% cellulose retention in the AC-pretreated poplar. The two step-treated poplar presented 86.6% glucose yield and 89.0% xylose yield by enzymatic hydrolysis with a cellulases loading of 7.2 m/g dry mass. Very high glucose (93.8%) and xylose (94.6%) yields were obtained with 14.3 mg cellulases/g dry mass. Both Tween 80 and β-glucosidase enhanced glucose yield of HPAC-pretreated poplar by alleviating the accumulation of cellobiose. Under the optimal conditions, 6.9 g XOS, 40.3 g glucose, and 8.9 g xylose were produced from 100 g poplar.

**Conclusions:**

The AC and HPAC pretreatment of poplar represented an efficient strategy to produce XOS and fermentable sugars with high yields. This two-step pretreatment was a recyclable benign and advantageous scheme for biorefinery of the poplar into XOS and monosaccharides.

**Electronic supplementary material:**

The online version of this article (10.1186/s13068-019-1423-x) contains supplementary material, which is available to authorized users.

## Background

Cellulosic biomass represents a renewable, low-cost resource that can be converted into biofuels and biochemicals [1, 2]. Poplar, as a fast growing, widely distributed, short rotation period crop, is considered as a suitable feedstock for this process [3, 4]. Enzymatic hydrolysis of poplar for the production of fermentable sugars, a key step for biofuels and biochemicals production, has been extensively investigated and different pretreatments, including ionic liquids, steam explosion, and acid/alkaline media, have been adopted to increase the accessibility of enzymes to polysaccharides [5–10]. These pretreatments are usually conducted with high temperature or long time [6]. Moreover, severe pretreatment could easily solubilize cellulose and xylan in poplar and made them hard to be recovered and utilized [7, 8, 10]. Therefore, efficiently utilization of polysaccharides in poplar still remains a challenge.

Xylooligosaccharides (XOS), derived from xylan in lignocellulosic biomass by enzymatic and/or chemical hydrolysis, have important prebiotic properties and show great potential application in medicinal, food, and health fields [11]. Recently, co-production of functional xylooligosaccharides and fermentable sugars from lignocellulosic materials by autohydrolysis, alkaline, and acid pretreatments has been reported [11–16]. Acetic acid (AC) pretreatment offered a green approach to effectively convert corncobs, viscose fibers, and poplar to XOS and fermentable sugars [14–16]. It has been reported that a good XOS yield of 45.91% was obtained from corncob by AC pretreatment at pH 2.7 and 150 °C for 30 min [14]. AC-pretreated poplar can produce a 36.0% XOS yield with 6.5% AC at 172 °C for 27 min with a clear increase (from 28.3 to 51.0%) of hydrolysis yield of AC-pretreated poplar by cellulases compared to poplar after autohydrolysis [15]. Normally, AC can be used to remove lignin with high temperature and high AC concentration [17]. However, after 6.5% AC pretreatment at 170 °C, the AC-pretreated poplar had high lignin content (> 30%) [15]. A large amount of lignin in poplar is unfavorable for enzymatic hydrolysis [18]. Thus, it is necessary to probe an effective method to alleviate lignin inhibition or to remove lignin to increase cellulases accessibility to cellulose in AC-pretreated poplar.

In this work, XOS and monosaccharides were produced from poplar by a combination of AC and hydrogen-peroxide/acetic acid (HPAC) pretreatments and enzymatic hydrolysis. Effects of AC concentration, temperature, time on XOS yields were investigated. After that, the AC-pretreated solid residues were then subjected to the HPAC process to remove lignin, aiming at enhancing the hydrolyzability of cellulose in poplar. The effects of Tween 80 and β-glucosidase on the hydrolysis of HPAC-pretreated poplar were also probed.

## Results and discussion

### AC pretreatment

#### Component analysis of AC-pretreated poplar

In the pretreatment of poplar with water (autohydrolysis) for 10, 30, and 50 min, the xylan content decreased from 17.4% to 16.5%, 11.0%, and 8.4%, respectively (Table 1). When the AC concentration was less than 5%, very high glucan retention (> 93%) was observed and the contents of glucan and lignin increased due to the removal of xylan by AC pretreatment. The highest acid insoluble lignin content (36.1%) was obtained from the poplar pretreated with 5% AC for 50 min. With the presence of 10% AC at 170 °C for 10 min, xylan and lignin were solubilized and the xylan and lignin removal increased from 6.8% and 0.3% to 40.2% and 6.0%, respectively. The data indicated that AC pretreatment was more effective than autohydrolysis pretreatment in xylan and lignin removal [18]. After pretreatment with 10% AC at 170 °C for 50 min, the xylan content decreased to 3.2% with a xylan removal of 87.8% and a lignin removal of 11.9%.

**Table 1:** Chemical compositions of poplar after AC pretreatment at 170 °C, expressed as percentage of dry mass (DM)

AC concentration	Treatment time (min)	Severity factor	Glucan (%)	Xylan (%)	Acid insoluble lignin (%)	Acid soluble lignin (%)	Solids recovery (%)	Removal		
								Glucan (%)	Xylan (%)	Lignin (%)
Raw		–	43.4 ± 0.0	17.4 ± 0.2	24.4 ± 0.3	3.5 ± 0.3	–	–	–	–
0%	10	3.1	43.9 ± 0.1	16.5 ± 0.5	25.1 ± 0.0	3.4 ± 0.3	98.1	0.8	6.8	0.3
5%	10	3.1	46.9 ± 0.9	14.8 ± 0.2	27.1 ± 0.3	3.0 ± 0.3	90.5	2.2	23.0	2.5
10%	10	3.1	52.3 ± 0.1	12.9 ± 0.1	29.9 ± 0.2	2.6 ± 0.3	80.7	2.7	40.2	6.0
0%	30	3.5	49.1 ± 0.0	11.0 ± 0.2	28.7 ± 0.1	2.7 ± 0.3	85.9	2.8	45.5	3.4
5%	30	3.5	54.9 ± 0.4	6.7 ± 0.5	33.4 ± 0.5	2.2 ± 0.3	74.7	5.5	71.3	4.9
10%	30	3.5	58.7 ± 0.6	5.1 ± 0.4	34.6 ± 0.1	2.0 ± 0.3	69.5	5.9	79.6	9.2
0%	50	3.8	53.6 ± 0.3	8.4 ± 0.4	32.6 ± 0.1	2.3 ± 0.3	77.0	4.8	62.8	3.7
5%	50	3.8	58.8 ± 0.2	4.3 ± 0.4	36.1 ± 0.1	2.0 ± 0.3	68.8	6.7	82.8	6.1
10%	50	3.8	57.6 ± 0.1	3.2 ± 0.2	35.6 ± 0.1	1.9 ± 0.3	65.6	12.9	87.8	12.0

Table 2 shows that 5% AC pretreatment for 30 min gave the highest XOS yield (55.8%), which was close to the yield of 51.5% (based on the xylan removed in pretreatment liquor) obtained in previous work [15]. However, the XOS yield from poplar was lower than that from corncob (62.2%, based on the xylan removed in pretreatment liquor) [14]. In this work, 5% AC and 30 min was chosen as the optimal AC conditions because it gave the highest XOS yield (55.8%) and acceptable loss of glucan (5.5%). After the optimal AC pretreatment, about 12.4 g xylan was removed in the pretreatment liquor from 100 g poplar and 6.9 g XOS and 4.6 g xylose were produced in the AC pretreatment liquor. The results indicated that some high DP oligomers were produced in AC pretreatment of poplar. The AC-pretreated poplar with optimal conditions contained 54.9 g glucan, 6.7 g xylan and 33.4 g lignin per 100 g of non-pretreated poplar, which corresponded to the removal of 71.3% of the original xylan and 4.9% of the original lignin (Table 1).

**Table 2:** The formations of xylose and XOS (DP 2–6) from poplar by the 0–10% AC pretreatment for 10–50 min

AC concentration (%)	Treatment time (min)	Xylose (%)	Xylooligosaccharide (%)					XOS yield (%, DP 2–6)
			Xylobiose	Xylotriose	Xylotetraose	Xylopentaose	Xylohexaose	
0	10	2.4	5.0	6.3	6.3	3.1	2.1	22.8
5	10	9.1	4.3	3.8	5.0	2.6	3.1	18.8
10	10	18.9	9.7	8.4	10.8	5.3	6.2	40.5
0	30	5.6	4.2	4.9	3.9	4.7	5.2	22.9
5	30	32.9	14.5	11.4	15.7	7.5	6.6	55.8
10	30	43.8	15.7	7.9	10.0	3.6	2.8	40.0
0	50	8.6	5.9	6.7	9.2	5.9	6.9	34.6
5	50	43.6	13.8	8.3	10.4	3.7	2.8	39.0
10	50	46.4	11.7	4.1	5.6	1.3	1.2	23.9

The XOS yields were based on the AC post-hydrolysis of pretreatment liquor

#### Characterization of AC-pretreated poplar

The XRD patterns of the non-pretreated and pretreated poplar samples showed that the crystalline structure of the cellulose in these poplar materials was unchanged after AC pretreatment and could be assigned as cellulose I (Additional file 1: Fig. S1) [19]. The crystallinity index (CI) indicates the relative amount of crystalline cellulose in the sample, and Table 3 demonstrated that the CI values of the poplar increased from 47.1% of raw poplar to 55.2% of AC-pretreated poplar (5% AC, 170 °C, 30 min). The AC pretreatment resulted in the increase in CI was due to the removal of xylan (Table 1). The removal of xylan can also be observed by FTIR spectroscopy (Additional file 1: Fig. S2). After the AC pretreatment, both the peak related to C=O groups associated with ester linkages between the lignin and hemicellulose at 1720 cm^−1^ and the peak due to the C–O–C stretching of the acetyl groups in hemicellulose at 1245 cm^−1^ decreased in intensity [20]. At the same time, the absorption bands at 1300–1600 cm^−1^ related to lignin increased in strength. The data here supported the selective removal of xylan by the AC pretreatment (Table 1).

**Table 3:** XPS, acetyl, hydrophobicity, and crystallinity index (%) analysis of non-pretreated and 5% AC-pretreated poplar

Treatment time	C1 (%)	C2 (%)	C3 (%)	O/C	Acetyl (%)	Hydrophobicity (L/g)	Crystallinity index (%)
Raw poplar	54.0	35.8	10.2	0.68	3.7 ± 0.2	0.37	47.1
10 min	56.0	34.0	10.0	0.37	5.7 ± 0.0	0.54	48.9
30 min	53.8	37.9	8.3	0.40	5.9 ± 0.0	0.49	55.2
50 min	49.4	44.5	6.1	0.41	5.7 ± 0.2	0.31	56.7

C1 corresponds to class of carbon that corresponds to carbon atoms bonded to carbon or hydrogen (C–C)

C2 corresponds to class of carbon atoms bonded to single non-carbonyl oxygen (C–O)

C3 corresponds to class of carbon atoms bonded to a carbonyl or two non-carbonyls (C=O or O–C–O)

The surface chemistry of biomass can be investigated by XPS [21] and a high O/C reflects higher cellulose and/or hemicellulose content, while a low O/C suggests the presence of more lignin [22]. The surface proportions of C and O atoms in the non-pretreated and pretreated poplar are provided in Table 3. In AC-pretreated poplar samples, C1, C2, and C3 carbon peaks were observed (Additional file 1: Fig. S3), which were in good agreement with the results obtained from corn stover [23, 24]. With the increase in pretreatment time from 10 to 50 min, the O/C ratio of AC-pretreated poplar increased from 0.37 to 0.41 and C1 peak intensity reduced from 56.0 to 49.4%, which indicated that surface lignin content decreased after AC pretreatment. The O/C ratio of AC-pretreated poplar was lower than that of raw poplar, possibly because of the removal of the extracts [21]. The C1 peak intensity of 10 min AC-pretreated poplar (56.0%) was higher than non-pretreated poplar (54.0%). The increase in C1 peak intensity indicated the increased surface lignin content. This result could be due to the lignin redeposition on the surface of poplar during AC pretreatment [22].

Hydrophobic interactions are regarded as the main driving force for the formation of non-productive binding between lignocellulose and cellulases [25]. The hydrophobicity values of the raw and pretreated poplar are presented in Table 3 and Additional file 1: Fig. S4. The hydrophobicity of the raw poplar was 0.37 g/L. As the AC treatment time increased from 10 to 50 min, the hydrophobicity of the poplar reduced from 0.54 to 0.31 g/L. However, the hydrophobicity of 5% AC and 30 min pretreated poplar was 0.49 g/L, which was still higher than that of the raw poplar. Hydrophobicity has been often characterized as a critical contributory factor to lignin inhibition of enzymatic hydrolysis [26] and cellulases adsorption onto lignin in pretreated substrates by hydrophobic interactions has been confirmed [25]. The higher hydrophobicity of AC-pretreated poplar indicated that the AC-pretreated poplar exhibited stronger cellulases adsorption capacity than the non-pretreated poplar [22].

The surfaces images of the raw and AC-pretreated poplar were characterized by SEM (Additional file 1: Fig. S5). The AC pretreatment resulted in less damage to the rigid structure of the raw poplar. Compared with the non-pretreated poplar, a plethora of spherical droplets was observed on the surfaces of the 5% and 10% AC-pretreated samples. These observations were in agreement with prior reports that the lignin droplets can be found on substrate after dilute acid pretreatment [27–30]. Lignin droplets probably led to a low hydrolysis yield of AC-pretreated poplar because the lignin droplets formed by acid pretreatment strongly inhibited cellulose hydrolysis in biomass by steric hindrance and non-productive adsorption of enzymes [31, 32].

#### Enzymatic hydrolysis of AC-pretreated poplar

**Fig. 1:**
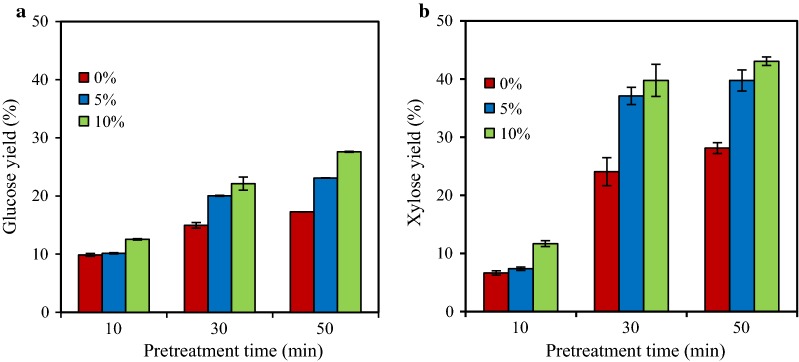
Enzymatic hydrolysis of AC pretreated poplar by cellulases. Glucose (**a**) and xylose (**b**) yield from AC (0–10% for 10–50 min) pretreated poplar by cellulases CTec2 (28.6 mg/g DM) at 50 °C and pH 5.0 for 48 h

Only 11.4% of glucose yield and 8.8% of xylose yield were obtained from the non-pretreated poplar with a cellulases loading of 28.6 mg/g DM. After pretreatment with 0% AC (autohydrolysis) for 10 and 50 min, glucose yields were 9.9% and 17.3%, respectively (Fig. 1a). In the enzymatic hydrolysis of poplar pretreated by 10% AC for 50 min, glucose yield increased to 27.6% and xylose yield reached 43.1% (Fig. 1b). With the increase in cellulases dosage up to 85.8 mg protein of cellulases/g DM, the glucose and xylose yields in AC-pretreated poplar (5%, 170 °C, 30 min) were 29.1% and 40.3%, respectively (Additional file 1: Fig. S6). The results here confirmed that hydrolysis yields of poplar pretreated by AC under the tested conditions were limited, which could be partially due to acetylation and the presence of high lignin content (> 25%) in the pretreated poplar [18, 33].

The 5% AC-pretreated poplar had higher acetyl content (more than 5.5%) than raw poplar (Table 3). It has been pointed out that pretreatment of lignocellulosic biomass with AC may result in the acetylation of its compounds such as cellulose and hemicellulose [31]. Enzymatic hydrolysis of the AC-pretreated poplar without any additional alkali pretreatment may not break the acetylated glucan and xylan and subsequently, affect their conversion to sugars [33]. In order to explore the effect of acetyl contents in AC-pretreated poplar on enzymatic hydrolysis, 1% (w/v) sodium hydroxide was used to treat AC-pretreated poplar (5% AC, 170 °C, 30 min) at 120 °C for 1 h with a solid loading of 10% (w/v). After sodium hydroxide pretreatment, the glucose yield of poplar by cellulases increased from 20.0 to 35.2%, indicating the increase in hydrolysis yield by alkali pretreatment. A similar phenomenon has also been observed in the hydrolysis of deacylated corncob and wood [14, 31, 34, 35]. In this work, such a low glucose yield (35.2%) was noticed in the hydrolysis of deacylated AC-pretreated poplar, which indicated that the acetylation of poplar might be not responsible for the main reason of the low hydrolysis yield of AC-pretreated poplar. Thus, the high residual lignin contents (25.1–36.1%) in the AC-pretreated poplar might be responsible for the relatively low hydrolysis yield [36, 37].

### HPAC pretreatment

#### Component analysis of HPAC-pretreated poplar

HPAC pretreatment is gaining more interest because of its highly effective removal of lignin [20, 38, 39]. In previous report, 98.08% of acid-insoluble lignin from pine wood was removed by 100% HPAC pretreatment at 80 °C for 2 h [38]. After 100% HPAC pretreatment at 80 °C for 3 h, about 90.3% lignin was removed and 86.0% enzymatic hydrolysis yield was obtained from Jerusalem artichoke [39]. It has been reported that 90.4% lignin removal and 84.0% glucose yield were observed from yellow poplar by HPAC pretreatment at 120 °C for 5 min [20]. In this work, after AC pretreatment, the poplar contained high lignin content (> 25%) and HPAC pretreatment was used for delignification of AC-pretreated poplar. With 40%, 60%, 80%, and 100% HPAC pretreatment of AC-pretreated poplar at 60 °C, the lignin content decreased from 33.4% to 22.4%, 15.3%, 4.3%, and 1.2%, respectively (Table 4). Pretreatment with 100% HPAC at 60 °C retained 90.9% glucan and removed 98.0% lignin and 23.5% xylan, indicating that HPAC pretreatment showed a powerful delignification capability. As the temperature increased from 40 to 80 °C, very high lignin removal (82.6–99.6%) was observed and the contents of glucan and xylan increased due to the removal of lignin by 100% HPAC pretreatment. Meanwhile, 2.5–13.0% glucan were lost as the HPAC pretreatment temperature increased from 40 to 80 °C (Table 4). The lowest lignin content (0.2%) in poplar was achieved with 100% HPAC at 80 °C for 2 h, which resulted in the highest glucan removal (13.0%). Our results indicated that HPAC pretreatment was highly effective for removing lignin from poplar, which was in good agreement with the results obtained from wood [38].

**Table 4:** Chemical compositions of HPAC-pretreated poplar, expressed as percentage of DM

HPAC concentration	Treatment temperature (°C)	Severity factor	Glucan (%)	Xylan (%)	Acid insoluble lignin (%)	Acid soluble lignin (%)	Solids recovery (%)	Removal		
								Glucan (%)	Xylan (%)	Lignin (%)
AC-pretreated poplar		–	54.9 ± 0.4	6.7 ± 0.5	33.4 ± 0.5	2.2 ± 0.3	–	–	–	–
40%	60	0.9	67.1 ± 1.4	8.4 ± 0.1	22.4 ± 0.2	0.4 ± 0.0	79.2	1.2	0.2	49.2
60%	60	0.9	76.8 ± 0.2	9.6 ± 0.1	15.3 ± 0.1	0.3 ± 0.0	68.6	4.1	1.5	70.0
80%	60	0.9	87.8 ± 0.2	9.2 ± 0.4	4.3 ± 0.1	0.1 ± 0.0	59.0	5.6	18.9	92.7
100%	40	0.3	79.9 ± 0.5	9.1 ± 0.3	9.0 ± 0.1	0.2 ± 0.0	67.0	2.5	8.4	82.6
100%	60	0.9	89.9 ± 0.1	9.2 ± 0.3	1.2 ± 0.0	0.1 ± 0.0	55.5	9.1	23.5	98.0
100%	80	1.5	89.9 ± 0.6	9.6 ± 0.2	0.2 ± 0.0	0.1 ± 0.0	53.1	13.0	24.0	99.6

The calculation of the removal of glucan, xylan and lignin in HPAC-pretreated poplar is based on the AC-pretreated poplar

#### Characterization of HPAC-pretreated poplar

After HPAC pretreatment (80% HPAC, 60 °C, 2 h), the CI value of the AC-pretreated poplar (5% AC, 170 °C, 30 min) increased from 55.2 to 64.2%. The HPAC pretreatments resulted in the increase in CI possibly because of the removal of lignin fractions (Tables 4, 5) [22]. Following the HPAC pretreatment, the FTIR absorption bands at 1300–1600 cm^−1^ related to lignin decreased (Additional file 1: Fig. S2). In addition, several absorption bands associated with glucan increased compared with those in the raw poplar spectrum. Specifically, the O–H stretch at 3330 cm^−1^ and the C–H stretch at 2900 cm^−1^ associated with glucan were stronger after the two-step pretreatment [20]. The results here indicated that HPAC pretreatment removed both xylan and lignin leaving a glucan-rich poplar (Table 5).

**Table 5:** XPS, acetyl, hydrophobicity, and crystallinity index (%) analysis of HPAC-pretreated poplar

Treatment temperature	HPAC concentration (%)	C1 (%)	C2 (%)	C3 (%)	O/C	Acetyl (%)	Hydrophobicity (L/g)	Crystallinity index (%)
AC-pretreated poplar		53.8	38.0	8.3	0.40	5.9 ± 0.0	0.49	55.2
60 °C	40	57.1	29.1	13.7	0.40	7.6 ± 0.1	0.27	57.7
60 °C	60	53.6	33.1	13.2	0.43	8.0 ± 0.0	0.14	63.0
60 °C	80	52.9	33.7	13.4	0.46	8.1 ± 0.0	0.07	64.2
40 °C	100	56.1	32.5	11.4	0.44	8.1 ± 0.2	0.21	60.1
60 °C	100	52.9	36.2	11.0	0.46	8.0 ± 0.2	0.06	64.9
80 °C	100	51.0	37.5	11.6	0.49	9.7 ± 0.1	0.06	68.5

It has been determined that in XPS analysis, the O/C ratios of different lignocellulose components decrease in the order of glucan > hemicellulose > lignin [21, 40]. After the HPAC pretreatment, the O/C ratio increased while the C1 peak intensity reduced (57.1–51.0%) as lignin was removed (Table 5). The O/C ratio (0.40) and C1 peak intensity (53.8%) of AC-pretreated poplar were very close to those of the HPAC-pretreated poplar. In addition, it was important to note that the content of acetyl group in the poplar increased when HPAC pretreatment became more severe (Table 5) possibly due to the formation of acetylated glucan and xylan during the pretreatment [33]. The acetylated glucan and xylan may change the O/C ratio of the substrates. Thus, the O/C ratio could not be used to evaluate the surface lignin content in HPAC-pretreated poplar. In addition, the hydrophobic acetate on the glucan surface could block the adsorption of cellulases [31].

**Fig. 2:**
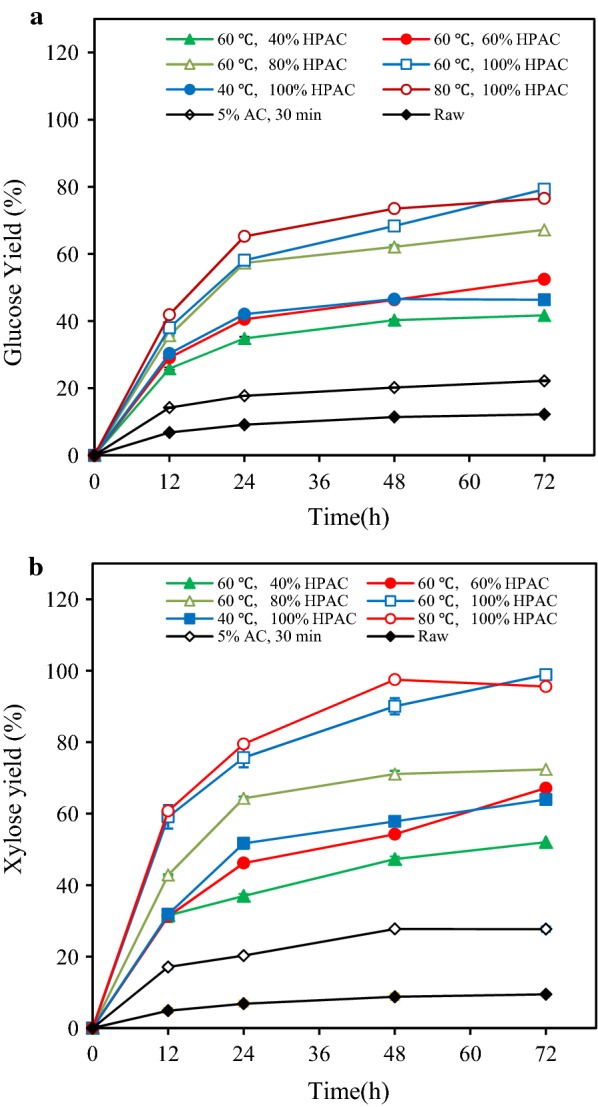
Glucose (**a**) and xylose (**b**) yields from 2% HPAC-pretreated poplar by CTec2 (28.6 mg/g DM) at 50 °C and pH 5.0 for 72 h. The HPAC pretreatment of poplar was based on the poplar pretreated with 5% AC at 170 °C for 30 min

The hydrophobicity of HPAC-pretreated poplar was lower than that of AC-pretreated poplar, and more severe HPAC pretreatment conditions reduced the hydrophobicity of the material (Table 5 and Additional file 1: Fig. S4). The main cause of this phenomenon was that the removal of lignin increased the relative contents of glucan in the remaining poplar. It has been reported that lignin is more hydrophobic than glucan, as hydrophobicity decrease as the O/C ratio increases [24]. Obviously, the removal of xylan and lignin had a pronounced effect on the hydrophobicity of the poplar and might affect hydrolysis yield of the pretreated poplar (Fig. 2).

Compared with the AC-pretreated material, the poplar after HPAC pretreatments showed more serious surface damage in the SEM photo (Additional file 1: Fig. S5). In addition, the lignin drops formerly seen on the AC-pretreated poplar disappeared after the HPAC treatment. Similar to pseudo-lignin, the re-deposited lignin droplets on the biomass surface were observed to have detrimental impacts on the enzymatic hydrolysis [27] and the removal of lignin droplets by HPAC might be helpful for enzymatic hydrolysis of AC-pretreated poplar.

#### Enzymatic hydrolysis of HPAC-pretreated poplar

After 40–100% HPAC pretreatment of the AC-pretreated poplar at 60 °C, the glucose yield enhanced from 20.0% to 41.7–79.2% (Fig. 2a), which could be due to the removal of lignin by HPAC and the increase in cellulases accessibility to cellulose [38]. When the pretreatment temperature increased from 40 to 80 °C, the glucose yield of 100% HPAC pretreated poplar increased from 46.3 to 76.5%. Meanwhile, 52.0–98.9% xylose yields were obtained from the HPAC-pretreated poplar (Fig. 2b). Such improved saccharification of HPAC-pretreated poplar could be attributed to the relatively high lignin removal (49.2% to 99.6%) during the pretreatment process (Table 4).

The observed 67.2% glucose and 72.4% xylose yields were unexpectedly low in light of the high degree of delignification (92.7%) which was achieved by 80% HPAC pretreatment at 60 °C for 2 h (Fig. 2a, Table 4). Only 76.5% glucose and 95.6% xylose yields were obtained from poplar with 99.6% lignin removal by HPAC pretreatment (100% HPAC, 80 °C for 2 h) (Fig. 2). A similar phenomenon has also been reported that the use of HPAC-pretreated poplar led to an unexpectedly enzymatic conversion (less than 80%) at a very low lignin content of 2.0% [41]. This phenomenon might due to the grafting of hydrophobic acetate on the cellulose surface blocked the adsorption of cellulases, thereby reducing the hydrolysis yield [31]. In order to explore the effect of acetyl content in HPAC-pretreated poplar on enzymatic hydrolysis, 0.1% (w/v) sodium hydroxide was used to treat HPAC-pretreated poplar (80% HPAC, 60 °C, 2 h) at 50 °C for 1 h with a solid loading of 10% (w/v) [35]. After sodium hydroxide treatment, the glucose yield of poplar increased from 67.2 to 74.4%. The data indicated that the acetylation might be one of reason to hinder the enzymatic hydrolysis of HPAC-pretreated poplar.

**Fig. 3:**
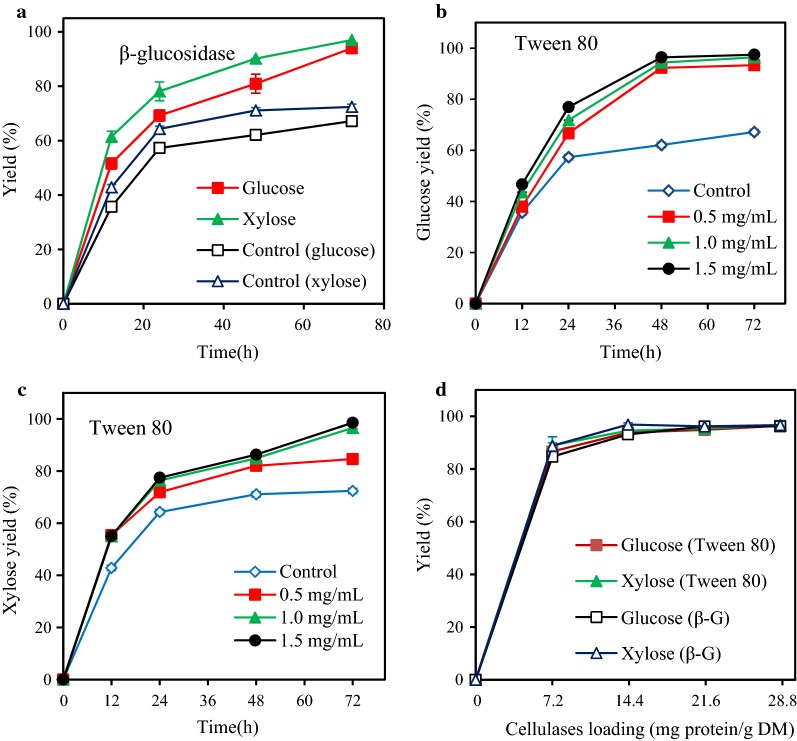
Effects of Tween 80 and β-glucosidase on the hydrolysis of 2% HPAC-pretreated poplar (80% HPAC, 60 °C) CTec2 (28.6 mg/g DM) at 50 °C and pH 5.0 for 72 h. **a** Effect of β-glucosidase (11.1 mg/g DM) on glucose and xylose yields. **b** Effect of Tween 80 (0.5–1.5 mg/mL) on glucose yield. **c** Effect of Tween 80 (0.5–1.5 mg/mL) on xylose yield. **d** Effect of Tween 80 (1.0 mg/mL) and β-glucosidase (11.1 mg/g DM) on the hydrolysis with different CTec2 loading

Interestingly, 2.7 mg/mL cellobiose was produced in the enzymatic hydrolysis of the HPAC-pretreated (80% HPAC, 60 °C, 2 h) poplar, which can strongly inhibit the action of cellobiohydrolase I and reduce enzymatic hydrolysis yield [42]. Supplementation of CTec2 cellulases with 11.1 mg protein of β-glucosidase per g DM improved the glucose and xylose yields from 67.2% and 72.4% to 94.0% and 97.0%, respectively (Fig. 3a).

Meanwhile, 0.05–0.24 mg/mL XOS (DP 2–6) were found in the enzymatic hydrolyte of HPAC-pretreated poplar. The inhibition of XOS to cellulases could also affect the hydrolysis yield of the HPAC-pretreated poplar [43]. Similar phenomenon has been reported that adding extra β-glucosidase with CTec2 improved the glucose and xylose yields of glycerol treated *Miscanthus* straw from 12.5% and 4.2% to 88.8% and 68.5%, respectively [44]. In this work, relatively high content of cellobiose in the hydrolysate of HPAC-pretreated poplar could inhibit the hydrolytic capacity of xylanase in CTec2 and decreased the xylose yield [45].

It has been reported that the use of surfactants in enzymatic hydrolysis can reduce the non-productive binding between lignin and cellulases and improve the enzymatic hydrolysis yield [46, 47]. 97.4% Glucose and 98.6% xylose yields were obtained from the HPAC-pretreated poplar after supplementation of 1 mg/mL Tween 80 with 28.6 mg protein of cellulases/g DM (Fig. 3b, c). After addition of Tween 80, the HPAC-pretreated poplar presented a remarkable hydrolysis yields of glucose (86.6%) and xylose (89.0%) with a cellulases loading of 7.2 mg/g DM (Fig. 3d), which could be explained by that Tween 80 increased the activity or stability of β-glucosidase and consequently alleviated the accumulation of cellobiose. Additionally, very high glucose (93.8%) and xylose (94.6%) yields also were obtained 14.3 mg protein of cellulases/g DM. Thus, employing Tween 80 instead of β-glucosidase was a viable mean to improve the hydrolysis yield. In this work, compared with previous reports on poplar 86.6% glucose yield with a cellulases loading (7.2 mg protein/g DM) or 93.8% glucose yield with 7.2 mg protein of cellulases/g DM and 1 mg/mL Tween 80 achieved satisfactory results [6, 7, 9, 15]. It has been reported that acetyl groups might inhibit the enzymes by interfering with the productive binding between cellulose and the catalytic domain of cellulases [31]. However, Tween 80 can change the ultrastructure of the substrate, making the cellulose more available to enzymatic attack and increase enzyme stability by reducing thermal denaturation or denaturation by shear forces [46]. Hence, this might be the reason that the Tween 80 increased enzymatic hydrolysis yield of the HPAC-pretreated poplar.

#### Comparison of different pretreatment methods of poplar

**Fig. 4:**
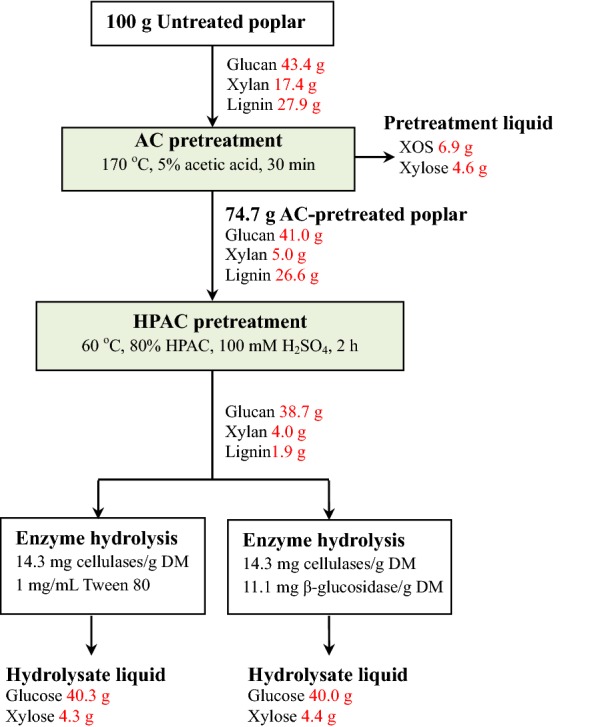
The overall mass balance for production of XOS and monosaccharides from poplar by a two-step AC and HPAC pretreatment

This work proposed a two-step pretreatment for the efficient production of XOS and fermented sugars from poplar and 6.9 g XOS, 40.3 g glucose and 8.9 g xylose could be produced from 100 g of poplar (Fig. 4). The AC and HPAC pretreated poplar presented remarkable hydrolysis yields of 86.6% glucose and 89.0% xylose at a cellulases loading of 7.2 mg/g DM and very high glucose (93.8%) and xylose (94.6%) yields were obtained with 14.3 mg protein of cellulases/g DM. The AC pretreatment and HPAC pretreatment were milder and more efficient than those associated with various typical poplar pretreatments (Table 6). Single step pretreatments such as steam explosion and those using alkaline peroxide or ionic liquids all require high temperatures and long treatment times and exhibit low enzymatic hydrolysis rates and poor conversion of certain components [6–8, 48, 49]. For example, only 60% enzymatic hydrolysis yield was obtained from poplar by a high temperature (210 °C) steam explosion pretreatment [7]. In the sulfuric acid and steam explosion pretreatments, the first step poplar pretreatment condition was 0.7% H_2_SO_4_ at room temperature overnight and the second step pretreatment condition was steam explosion at 190 °C for 10 min and finally only 70% glucose yield was obtained by 72 h enzymatic hydrolysis with loading 32 mg cellulases/g DM [9]. Additionally, two-step poplar pretreatments such as sodium hydroxide and sodium sulfate, white-rot and sodium hydroxide, sulfuric acid and steam explosion, sulfur dioxide steam explosion and ethanol can improve poplar hydrolysis yield, but the associated conditions are still very severe [5, 9, 10]. In this work, xylan in poplar was efficiently used for XOS production and glucan was almost completely hydrolysis. After AC pretreatment, the AC concentration in pretreatment liquor (5.2 mg/mL AC, 170 °C, 30 min) was 4.7 mg/mL (Additional file 1: Table S1). AC in the pretreatment liquor can be recycled by liquid–liquid extraction [50]. During the HPAC pretreatment, the AC was consumed and peroxyacetic acid was formed. Pressure shift distillation coupled with a separation process was capable to separate AC and peroxyacetic acid in HPAC pretreatment liquor [51, 52]. The two-step pretreatment of AC and HPAC provided a preferable feature to prepare XOS and monosaccharides from poplar with relatively mild conditions, showing great potential application in industrial production of XOS, biofuels, and biochemicals from biomass.

**Table 6:** The enzyme hydrolysis of pretreated poplar by different methods

Raw material	Pretreatment method and conditions	Enzyme hydrolysis condition	Glucose yield (%)	References
Transgenic poplar	[C_2_C_1_Im][OAc] at 160 °C for 3 h	10 mg protein of cellulases/g DM in 72 h	< 35.0	[6]
	[Ch][Lys] at 140 °C for 1 h	10 mg protein of cellulases/g DM in 72 h	< 40.0	
	[TBA][OH] at 70 °C for 3 h	10 mg protein of cellulases/g DM in 72 h	< 30.0	
Poplar	Steam explosion at 210 °C for 4 min	15 FPU cellulases/g DM in 72 h	< 63.0	[7]
Sacrau poplar	2% NaOH with 1% H_2_O_2_ at 160 °C for 2 h	20 FPU cellulases/g DM and Tween 80 in 72 h	88.2	[12]
Poplar	6.5% acetic acid at 172 °C for 27 min	20 FPU cellulases/g cellulose in 108 h	< 51.0	[15]
Poplar	0.1 M H_2_SO_4_ at 160 °C for 35 min	112.5 mg protein of cellulases + 37.5 mg protein of xylanases/g glucan + xylan in 72 h	< 60	[48]
Poplar	Hot water at 180 °C for 12 min	15 mg protein of cellulases/g glucan in 120 h	60.0	[49]
Hybrid poplar	First step: 8% NaOH at 90 °C for 10 h Second step: 4% Na_2_SO_3_ at 180 °C for 60 min	15 FPU cellulases/g DM in 72 h	94.5	[5]
Poplar	First step: 0.7% H_2_SO_4_ at room temperature overnight Second step: steam explosion at 190 °C for 10 min	32 mg cellulases protein/g DM in 72 h	70	[9]
Poplar	First step: SO_2_ steam explosion at 170 °C for 15 min Second step: ethanol with 1% H_2_SO_4_ at 170 °C for 60 min	5 FPU cellulases/g cellulose in 72 h	85	[10]
Poplar	First step: 5% AC 170 °C for 30 min Second step: 80% HPAC at 60 °C for 2 h	7.2 mg protein of cellulases/g DM and Tween 80 in 72 h	86.6	This study
Poplar	First step: 5% AC 170 °C for 30 min Second step: 80% HPAC at 60 °C for 2 h	14.3 mg protein of cellulases/g DM and Tween 80 in 72 h	93.8	This study

## Conclusions

The AC pretreatment of poplar solubilized the most xylan in poplar and gave rise to a 55.8% XOS yield. The HPAC pretreatment presented 49.2–99.6% lignin removal of AC-pretreated poplar and improved the glucose yield from 20.0% to above 90% with a dosage of 14.3 mg protein of cellulases/g DM and 11.1 mg protein of β-glucosidase/g DM or 1 mg/mL Tween 80. The two-step pretreatment offered an approach to effectively converting 100 g poplar to 6.9 g XOS and 49.2 g monosaccharides. The AC and HPAC two-step pretreatment of poplar for producing XOS and fermentable sugars could facilitate means to develop an economically viable biorefinery process.

## Methods

### Materials

Poplar used in this work was kindly supplied by Prof. Yong Xu from Nanjing Forestry University. The material was air dried for 3 days, followed by pulverization to obtain a powder with an 80 mesh particle size (≤ 0.178 mm), having a moisture content of 9.40%. The contents of glucan, xylan, acid insoluble lignin and acid soluble lignin in the raw poplar were 43.38%, 17.36%, 24.43%, and 3.50%, respectively, determined using a method published by the National Renewable Energy Laboratory [53].

Cellic CTec2 (Novozymes A/S, Bagsværd, Denmark) had an activity of 123.0 filter paper units (FPU)/mL (176.2 mg protein/mL) determined according to the International Union of Pure and Applied Chemistry standard assay [54]. The Novozyme 188 (β-glucosidase) was determined to be 8451 nkat/mL (187.9 mg protein/mL) as described previously [55]. The enzyme protein was quantified by the Lowry method using bovine serum albumin (Sigma-Aldrich, St. Louis, MO, USA) as the standard [56]. Xylobiose, xylotriose, xylotetraose, xylopentaose, and xylohexaose were purchased from Megazyme (Wicklow, Ireland).

### AC pretreatment

The AC pretreatment was performed in a sealed, Teflon-lined stainless steel autoclave (HT-100H-316L, Anhui Kemi Machinery Technology Co., Ltd., Anhui, China) heated in a Constant Temperature Oil Bath (HH-SB, Jinhua Wenhua Equipment and Instrument Co., Ltd., Zhejiang, China). The AC pretreatment conditions were referred to a previous report [15]. The system was heated about 30 min from 25 to 170 °C (5 °C/min). AC concentrations of 0%, 5% (5.2 mg/mL), or 10% (v/v) (10.5 mg/mL) were used to treat poplar samples with a solid loading of 10% (w/v). The process was performed at 170 °C for 10, 30 or 50 min. Each experiment was carried out in triplicate. Following the reaction, the sealed tank was taken out from oil bath and immediately cooled down to room temperature with tap water in 30 min. The pretreatment liquor was separated from the slurry through vacuum filtration. Part of pretreatment liquor was used to determine the XOS concentration. The solid was recovered by filtration and washed repeatedly with distilled water until the wash water had a neutral pH, then stored at − 20 °C for chemical composition analysis and enzymatic hydrolysis.

### HPAC pretreatment

A sample of poplar pretreated by 5% AC at 170 °C for 30 min was employed for the subsequent HPAC pretreatment. The HPAC solution was prepared by mixing hydrogen peroxide (30%, w/w) and acetic acid (99%, w/w) at a ratio of 1:1 (v/v) [38]. To investigate the effects of temperature on HPAC pretreatment, AC-pretreated poplar were pretreated with 100% (v/v) HPAC at temperatures of 40 °C, 60 °C, and 80 °C. The effect of HPAC concentration (40–100%, v/v) on pretreatment was investigated at 60 °C. All pretreatments were preformed for 2 h at a solid to liquid ratio of 1:10 (w/v) with 100 mM H_2_SO_4_ as a catalyst. The solid residues were separated by filtration and washed extensively with distilled water until the wash water had a neutral pH, then stored at − 20 °C for chemical composition analysis and enzymatic hydrolysis.

### Enzymatic hydrolysis

Hydrolysis of the raw and treated poplar was performed in test tubes (601051-1, Biosharp, Hefei, China) with a 3 mL working volume in a 50 mM sodium citrate buffer (pH 5.0) at 50 °C and 200 rpm [53]. NaN_3_ (0.02% w/v) was added to the hydrolysis broth to prevent bacterial growth. The AC-pretreated poplar loading in each reaction mixture was 2% (w/v), with a cellulases loading of 14.3, 28.6, 43.0, 57.3, 71.3, 85.8 mg protein (10, 20, 30, 40, 50, 60 FPU)/g DM. The cellulases loading in hydrolysis of HPAC-pretreated poplar was 7.2, 14.3, 21.5, 28.6 mg protein (5, 10, 15, 20 FPU)/g DM. The β-glucosidase loading was 500 nakt (11.1 mg protein)/g DM for hydrolysis of HPAC-pretreated poplar. The concentration of Tween 80 in hydrolysate was 0.5–1.5 mg/mL. Two tubes of replicate test were withdrawn at intervals ranging from 12 to 72 h and boiled for 10 min to stop the enzymatic hydrolysis. Enzymatic hydrolysate was boiled for 10 min to stop the enzymatic hydrolysis. After cooling, the samples were separated by centrifugation (10,000×*g*, 10 min) and the cellobiose, XOS, glucose, and xylose in the supernatants were analyzed. Two replicate tests were performed for all hydrolysis experiments and average values are presented.

### Characterization of solid residues

The crystallinity index (CI) of each raw and treated poplar specimen was determined by XRD using a Rigaku D/max-3 C instrument (Rigaku Corporation, Japan) [19]. The CI was determined using the equation:

1 $${\text{CI\% }} = \frac{{I_{002} - I_{\text{am}} }}{{I_{002} }} \times 100{\text{\% }}$$

where *I*_002_ is the maximum intensity of the diffraction at approximately 22.5° that corresponds to crystalline regions and *I*_am_ is the minimum intensity of the peak at approximately 18.0° that corresponds to amorphous regions.

Chemical functional groups were characterized using a Nicolet iS10 FTIR spectrophotometer (Thermo Fisher, USA), acquiring spectra over the range of 4000–400 cm^−1^ and adding 32 scans to generate each spectrum. The background spectrum of the diamond window without a sample was subtracted from each sample spectrum.

XPS analyses were performed based on the method reported by Kumar and Montplaisi to determine the atomic compositions and chemical environments of the poplar sample surfaces [24, 57]. The hydrophobicities of the poplar samples were determined via the method previously described [58, 59].

The surface morphologies of the raw and pretreated poplar specimens were observed by SEM (JSM 6360LV, Jeol, Japan) with an acceleration voltage of 10 kV. The samples were coated with gold to make them conductive before imaging and images were acquired at 1000, 2000 and 5000× magnification.

### Analytical methods

The acetyl contents of poplar solids were determined as per NREL LAP002 using glacial acetic acid as a calibration standard [53].

The calculation of XOS (DP 2-6) yields was based on the removed xylan in the pretreatment liquor. Total oligosaccharide contents of the AC pretreatment liquors were determined indirectly after quantitative acid hydrolysis with 4% H_2_SO_4_ at 121 °C for 1 h, according to NREL/TP-510-42623. XOS (DP 2-6) was analyzed using high performance anion exchange chromatography coupled with pulsed amperometric detection (Dionex ICS-5000). The analysis employed 0.1 M NaOH and 0.5 M NaOAc containing 0.1 M NaOH as the mobile phases, at a flow rate of 0.3 mL/min in conjunction with a CarboPac PA200 anion exchange column [60].

Acetic acid, cellobiose, and monosaccharides were analyzed by high-performance liquid chromatography (Agilent 1260), using an Aminex Bio-Rad HPX-87H column and a refractive index detector together with 5 mM H_2_SO_4_ as the mobile phase at a flow rate of 0.5 mL/min. All samples were diluted with Milli-Q water prior to analysis. Two replicate assays were performed for each sample, and average values are presented.

### Calculations

The pretreatment severity factor was used to monitor and compare the pretreatment severity [24, 61].

2 $${\text{Severity}}\;\;{\text{factor}} = \log \left[ {t\; *\;\exp \left( {\frac{{T_{\text{i}} - T}}{14.75}} \right)} \right]$$

where *t* refers to pretreatment time (min), *T*_i_ is the reaction temperature (°C), and *T* is the reference temperature of 100 °C.

The percent recovery and percent removal of each component were calculated by the following formula [62]:

3 $${\text{Soild recovery }}\left( {\text{\%}} \right) = \frac{{W_{{{\text{after}}\;{\text{pretreatment}}}} }}{{W_{\text{before pretreatment}} }} \times 100$$

where *W*_before pretreatment_ and *W*_after pretreatment_ were the weight of poplar before and after pretreatment.

4 $${\text{Removal }}\left( {\text{\%}} \right) = 1 - \frac{{W_{\text{component after pretreatment}} }}{{W_{\text{component before pretreatment}} }} \times 100$$

where *W*_component_ _before pretreatment_ and *W*_component_ _after pretreatment_ were the weight of the components (cellulose, xylan and, lignin) in poplar before and after pretreatment (g).

The xylose and XOS (DP 2 to 6) yields from AC pretreatment liquor were calculated based on the following equations [16, 63]:

5 $${\text{Xylose yield}}_{\text{AC pretreatment liquor }} \left( {\text{\%}} \right) = \frac{{ {\text{Xylose in AC pretreatment liquor }}\left( {\text{g}} \right) \times 0.88}}{{{\text{Removed xylan in AC pretreatment liquor }}\left( {\text{g}} \right)}} \times 100$$

6 $${\text{XOS yield }}_{\text{AC pretreatment liquor }} \left( {\text{\%}} \right) = \frac{{ {\text{XOS }}\left( {{\text{DP }}2 - 6} \right) {\text{in AC pretreatment liquor }}\left( {\text{g}} \right)}}{{{\text{Removed xylan in AC pretreatment liquor }}\left( {\text{g}} \right)}} \times 100$$

The yields of glucose, xylose, cellobiose, and XOS (DP 2 to 6) were calculated based on the following equations [16]:

7 $${\text{Glucose yield }}\left( {\text{\%}} \right) = \frac{{{\text{Glucose in enzymatic hydrolysate}} \times 0.9}}{\text{Theoretical amount of glucan in pretreated poplar}} \times 100$$

8 $${\text{Cellobiose yield }}\left( {\text{\%}} \right) = \frac{{{\text{Cellobiose in enzymatic hydrolysate}} \times 0.9}}{\text{Theoretical amount of glucan in pretreated poplar}} \times 100$$

9 $${\text{Xylose yield }}\left( {\text{\%}} \right) = \frac{{{\text{Xylose in enzymatic hydrolysate}} \times 0.88}}{\text{Theoretical amount of glucan in pretreated poplar}} \times 100$$

10 $${\text{XOS yield}}\;\left( {\text{\%}} \right) = \frac{{{\text{XOS}}\left( {{\text{DP }}2 - 6} \right) {\text{in enzymatic hydrolysate}}}}{\text{Theoretical amount of xylan in pretreated poplar}} \times 100$$

## Additional file

**Additional file 1: Table S1.** The AC concentration and recovery of AC pretreatment liquor. **Figure S1.** XRD analysis of raw and two steps treated**. Figure S2.** FT-IR spectrum of raw and two steps pretreated poplar. **Figure S3.** XPS analysis of raw and two steps pretreated poplar. **Figure S4.** Hydrophobicity of raw and two steps pretreated poplar. **Figure S5.** SEM analysis of raw and two steps pretreated poplar. **Figure S6.** Effect of CTec2 loading on the hydrolysis of poplar (2%) pretreated by AC (5%, 170 °C, 30 min) for 48 h.

## Abbreviations

AC: acetic acid
HPAC: hydrogen peroxide–acetic acid
XOS: xylooligosaccharides
DM: dry mass
FT-IR: Fourier transform-infrared
SEM: scanning electron microscopy
XRD: X-ray diffraction
XPS: X-ray photoelectron spectroscopy
FPU: filter paper unit
