# Supplementary material for: Production of xylooligosaccharides and monosaccharides from poplar by a two-step acetic acid and peroxide/acetic acid pretreatment
Source: Biotechnol Biofuels. 2019 Apr 15;12:87. doi: 10.1186/s13068-019-1423-x (PMC6463647; doi:10.1186/s13068-019-1423-x)
Supplement: Supplementary file 1 — Additional file 1: Table S1. The AC concentration and recovery of AC pretreatment liquor. Figure S1. XRD analysis of raw and two steps treated. Figure S2. FT-IR spectrum of raw and two steps pretreated poplar. Figure S3. XPS analysis of raw and two steps pretreated poplar. Figure S4. Hydrophobicity of raw and two steps pretreated poplar. Figure S5. SEM analysis of raw and two steps pretreated poplar. Figure S6. Effect of CTec2 loading on the hydrolysis of poplar (2%) pretreated by AC (5%, 170 °C, 30 min) for 48 h. [file 13068_2019_1423_MOESM1_ESM.docx]

**Production of xylooligosaccharides and monosaccharides from poplar by a two-step acetic acid and peroxide/acetic acid pretreatment**

Peiyao Wen^1^, Tian Zhang^1^, Jinye Wang^1^, Zhina Lian^2^, Junhua Zhang^1, 2^*

Corresponding author. Tel.: +86-13892883052; fax: +86-29-87082216.

E-mail address: junhuazhang@nwafu.edu.cn (J. Zhang)

Peiyao Wen, e-mail address: wenpeiyao@nwafu.edu.cn

Tian Zhang, e-mail address:15002906131@163.com

Jinye Wang, e-mail address: wjy6520@nwafu.edu.cn

Zhina Lian, e-mail address: [zhinalian@163.com](mailto:zhinalian@163.com)

**Table S1** The AC concentration of AC pretreatment liquor.

| AC concentration before pretreatment ( mg/mL) | Treatment time | AC concentration after pretreatment (mg/mL) |
| --- | --- | --- |
|  |  |  |
| 0.0 | 10 min | 0.21±0.00 |
| 5.2 | 10 min | 3.49±0.08 |
| 10.5 | 10 min | 7.60±0.11 |
| 0.0 | 30 min | 0.22±0.04 |
| 5.2 | 30 min | 4.68±0.23 |
| 10.5 | 30 min | 8.41±0.21 |
| 0.0 | 50 min | 0.23±0.09 |
| 5.2 | 50 min | 4.70±0.14 |
| 10.5 | 50 min | 8.41±0.16 |

**\**

**Figure S1** XRD analysis of raw and two steps pretreated poplar.

**Figure S2** FT-IR spectrum of raw and two steps pretreated poplar**.**

First step pretreatment

Second step pretreatment

**Figure S3** XPS analysis of raw and two steps pretreated poplar.

**Figure S4** Hydrophobicity of raw and two steps pretreated poplar.

**
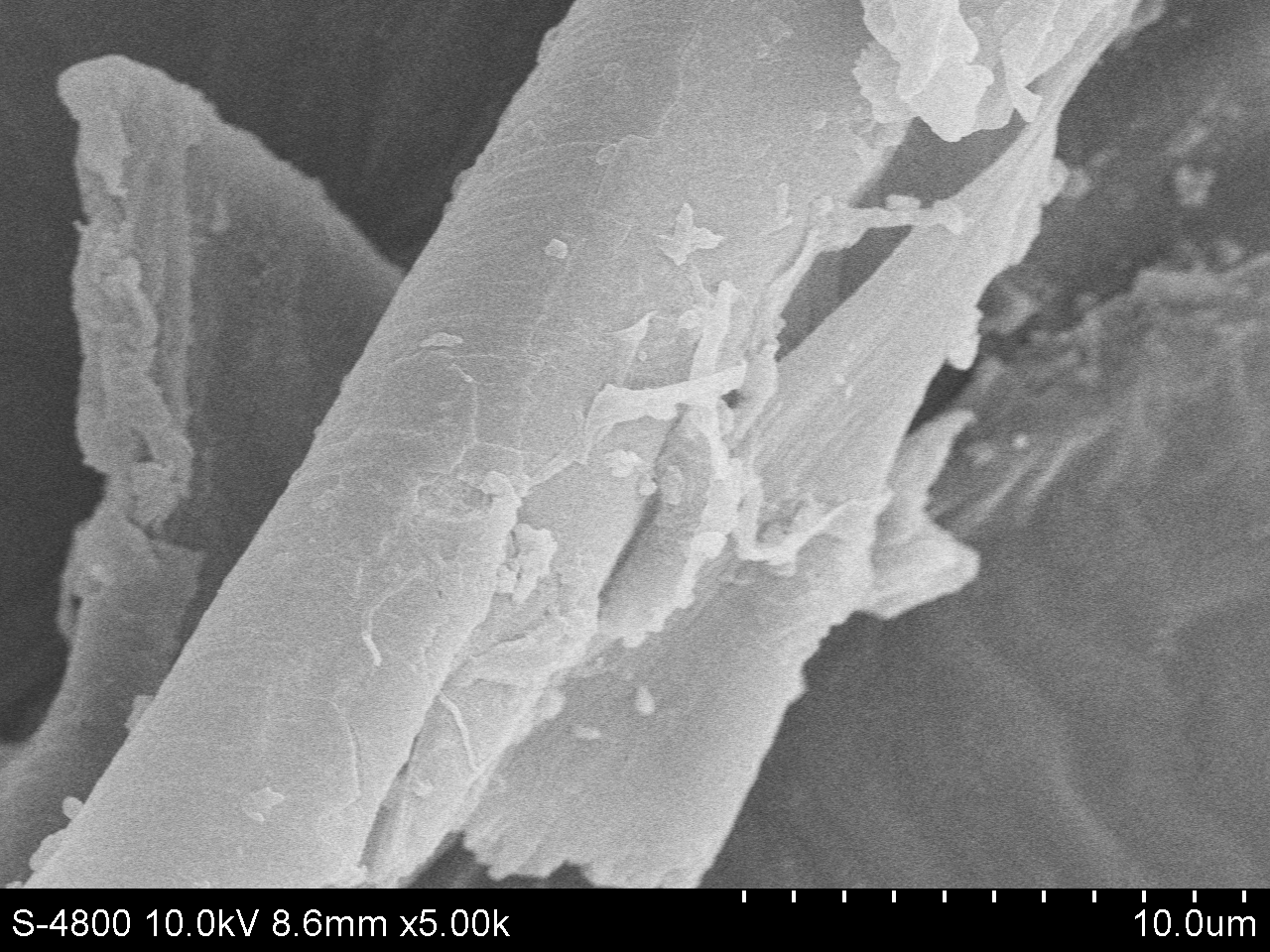
**

**Raw**

**The first step pretreatment**


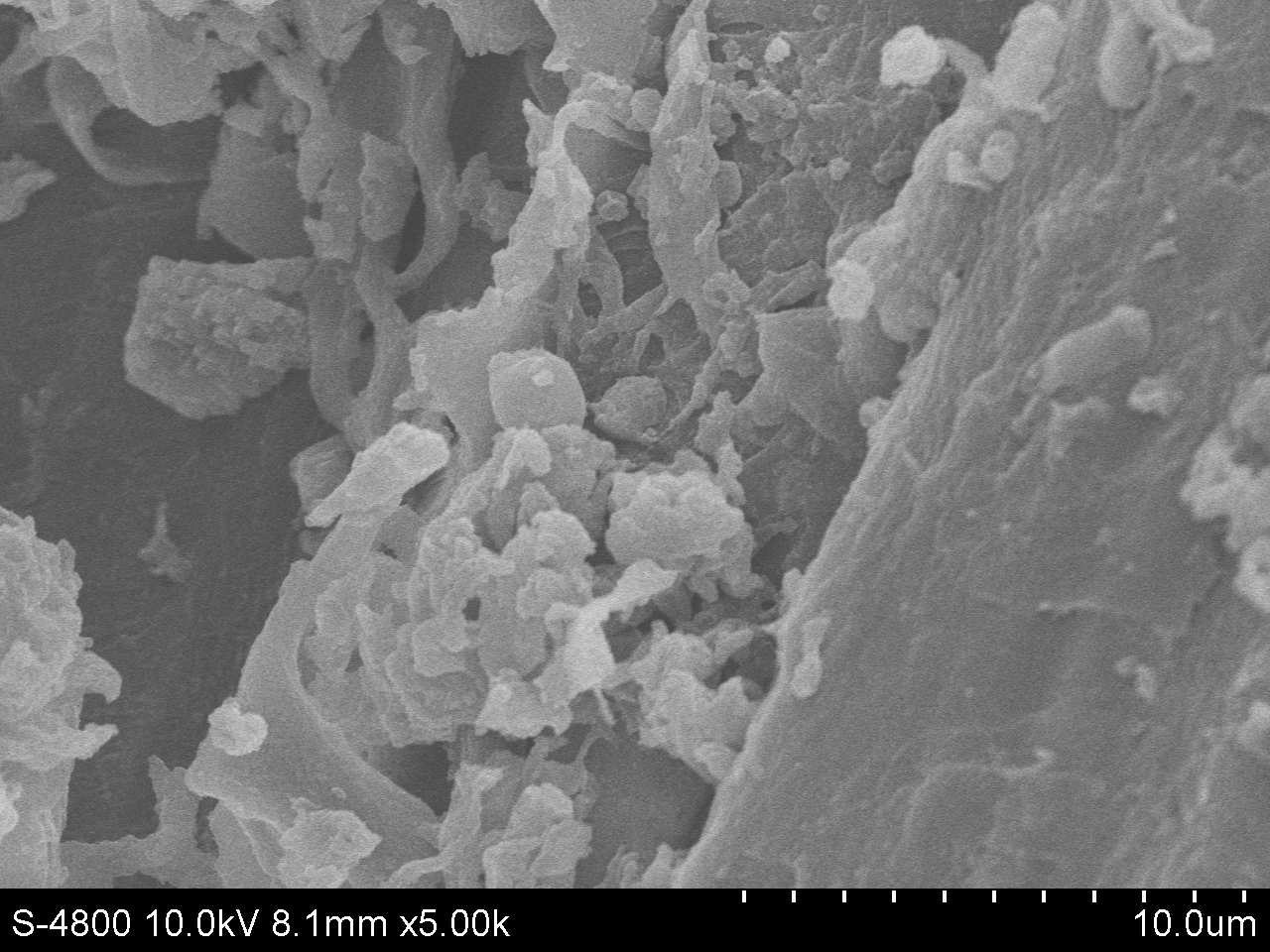



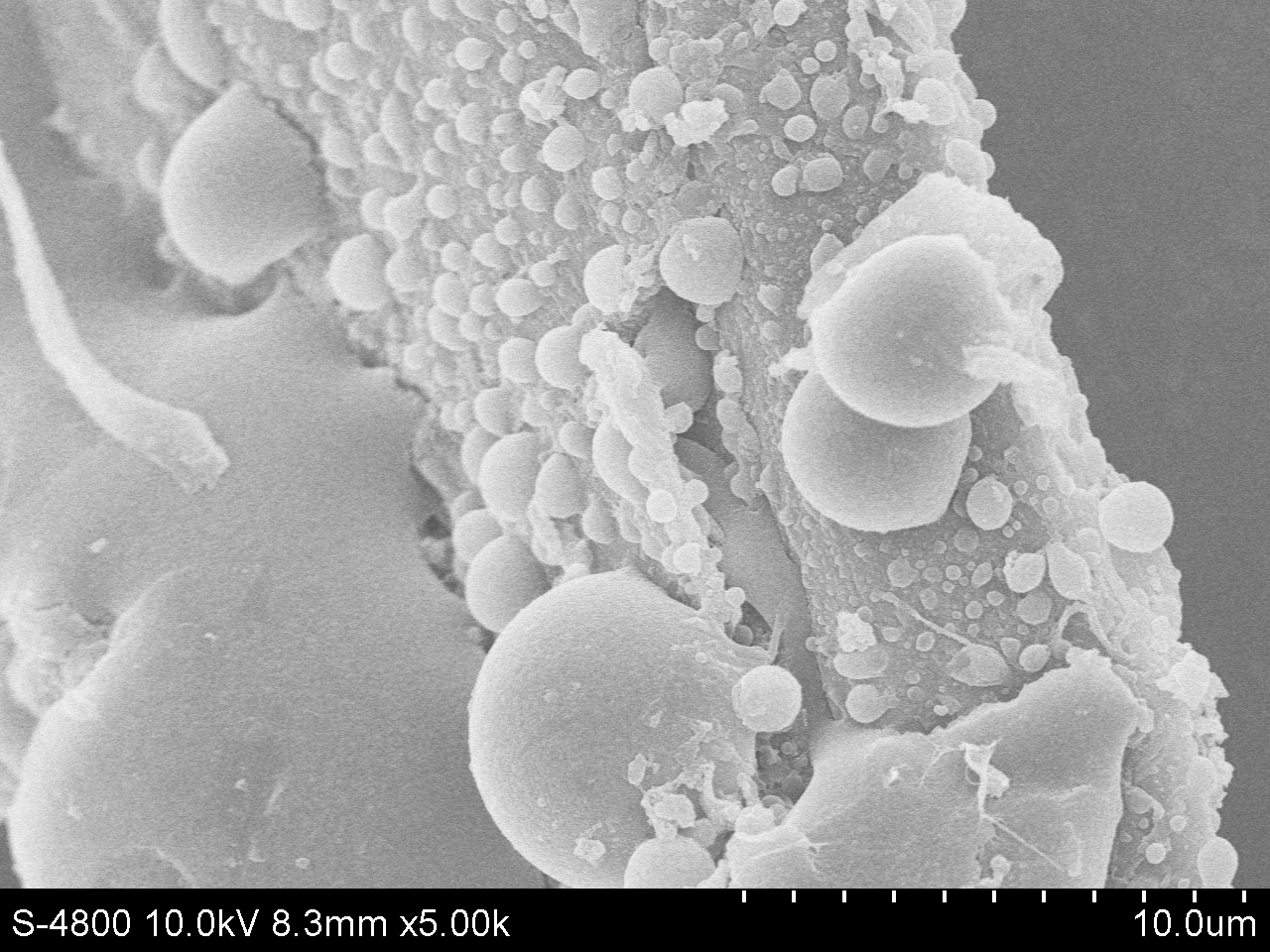


0% AC, 30 min

5% AC, 30 min

10% AC, 30 min

**The second step pretreatment**


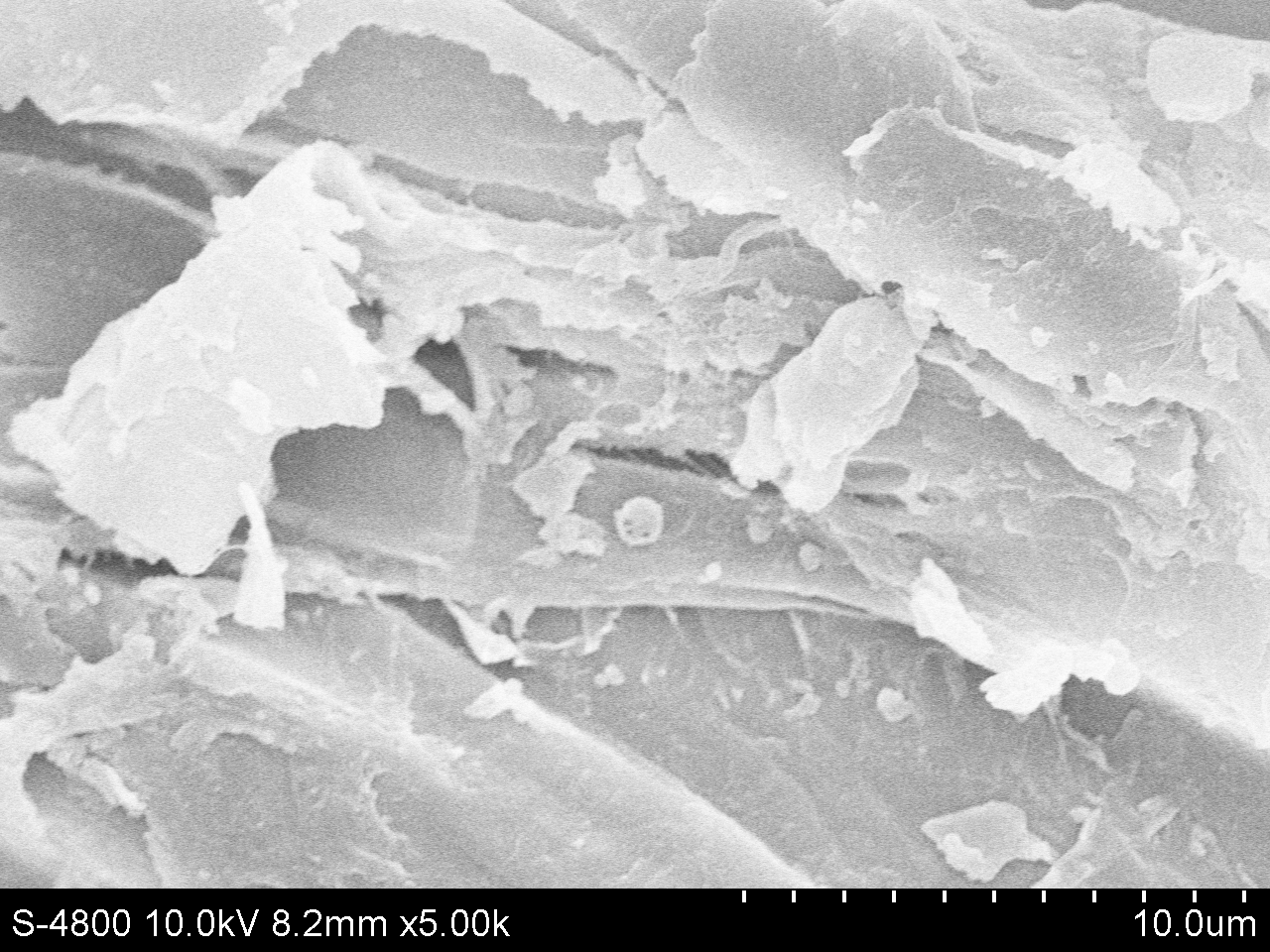

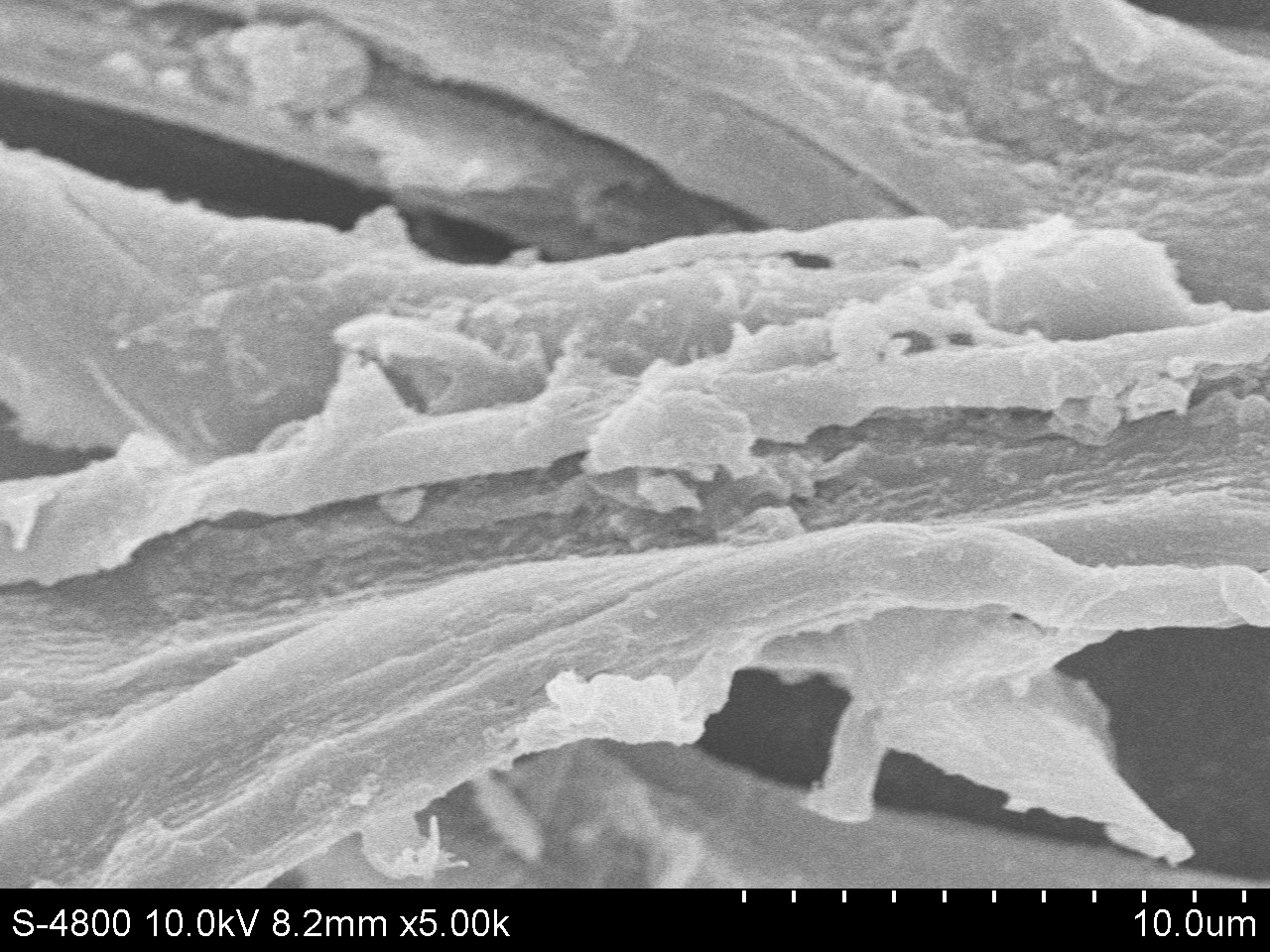

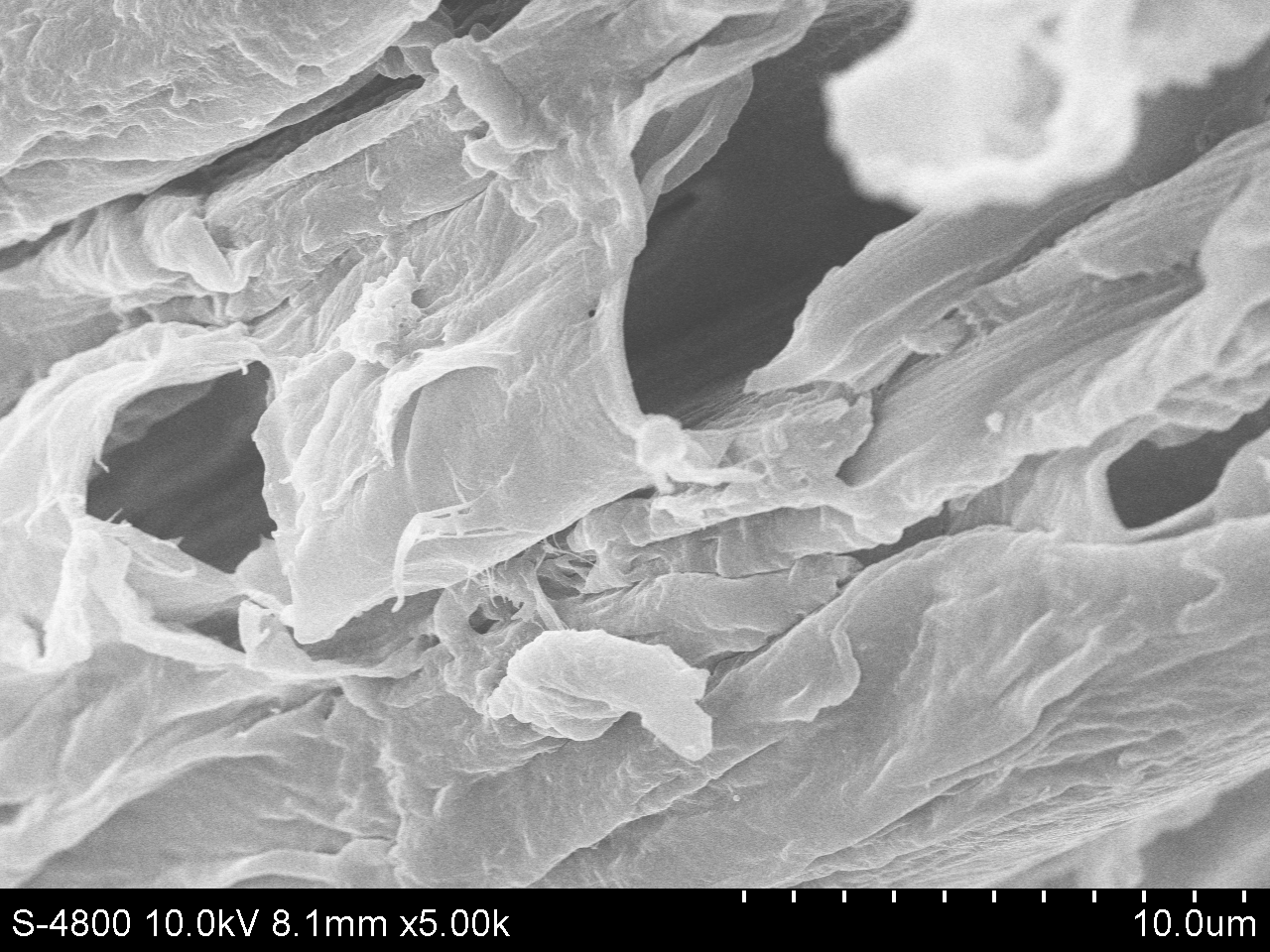


40% HPAC, 60 °C

80% HPAC, 60 °C

60% HPAC, 60 °C

**Figure S5** SEM analysis of raw and two steps pretreated poplar.

**Figure S6** Effect of CTec2 loading on the hydrolysis of poplar (2%) pretreated by AC (5%, 170°C, 30 min) for 48 h**.**
